# Transcriptome dataset of two *Pistacia* species: *Pistacia chinensis* and *Pistacia weinmannifolia*

**DOI:** 10.1016/j.dib.2023.110002

**Published:** 2023-12-22

**Authors:** Azeez Bimpe Suliyat, Dewi Komang Anggita, Hee Soo Yang, Sang-Woo Lee, Wan Yi Li, Sang-Ho Choi, Ki-Young Choi, Jong-Kuk Na

**Affiliations:** a Department of Agriculture and Industries, Graduate School, Kangwon National University, Chuncheon 24341, South Korea; b International Biological Material Research Center, KRIBB, Daejeon 34141, South Korea; c Institute of Medicinal Plants, Yunnan Academy of Agricultural Sciences, Kunming, Yunnan 650200, PR China; d Department of SmartFarm and Agricultural Industries, Kangwon National University, Chuncheon, Kangwon 24341, South Korea

**Keywords:** Ethnobotanical, Illumina HiSeq2500, RNA-seq, Small tree, Transcriptome shotgun assembly

## Abstract

*Pistacia chinensis* and *Pistacia weinmannifolia* are small trees and are distributed in East Asia, in particular China. The data on *P. chinensis* presented in this article is associated with the research article, “DOI: 10.5010/JPB.2019.46.4.274” [1]. Both *P. chinensis* and *P. weinmannifolia* have long been used as ethnobotanical plants to treat various illnesses, including dysentery, inflammatory swelling, rheumatism, liver diseases, influenza, lung cancer, etc. Many studies have been carried out to delve into the pharmaceutical properties of these *Pistacia* species using plant extracts, but genomic studies are very rarely performed to date. To enrich the genetic information of these two species, RNA sequencing was conducted using a pair-end Illumina HiSeq2500 sequencing system, resulting in 2.6 G of raw data from *P. chinensis* (Accession no: SRR10136265) and 2.7 G bases from *P. weinmannifolia* (Accession no: SRR10136264). Transcriptome shotgun assembly using three different assembly tools generated a total of 18,524 non-redundant contigs (N50, 1104 bp) from *P. chinensis* and 18,956 from *P. weinmannifolia* (N50, 1137 bp). The data is accessible at NCBI BioProject: PRJNA566127. These data would be crucial for the identification of genes associated with the compounds exerting pharmaceutical properties and also for molecular marker development.

Specifications Table

**Table:** 

Subject	Agronomy and Crop Science
Specific subject area	Transcriptome analysis of medicinal plants
Data format	Raw data: fastq sequencing files Processed data: transcriptome assembled sequences after filtering raw data
Type of data	Table, Figure
Data collection	RNA sequencing was performed by the Illumina Hiseq 2500 (Illumina, USA). NGS tool kits and Ion-Torrent server were used for filtering to remove low-quality reads. Filtered quality reads were used for transcriptome sequence assembly using three assembling tools; CLC Genomics Workbench (ver. 3.7.1), Velvet-Oases (ver. 1.1.04-ver. 0.1.21), and Trinity (release 20110519).
Data source location	Kangwon National University, Chuncheon, Gangwon Province, South Korea
Data accessibility	Repository name: NCBI BioProject BioProject data accession number: PRJNA566127 BioProject data accession: https://www.ncbi.nlm.nih.gov/bioproject/?term=PRJNA566127 NCBI Transcriptome Shotgun Assembly Project (TSA) depository accessions: GKQH00000000 (*P. chinensis*), GKQG00000000 for (*P. weinmannifolia*) TSA accession: https://www.ncbi.nlm.nih.gov/nuccore/GKQG00000000 : https://www.ncbi.nlm.nih.gov/nuccore/GKQH00000000
Related research article	Choi KY, Park DH, Seong ES, Lee SW, Hang J, Yi LW, Kim JH, Na JK (2019) Transcriptome analysis of a medicinal plant, *Pistacia chinensis*. J. Plant. Biotechnol. 46(4): 274–281 (https://www.kspbtjpb.org/journal/view.html?volume=46&number=4&spage=274)

## 1 Value of the Data

• The data provides transcripts as the first transcriptome reference for *P. weinmmanifolia*, and as for *P. chinensis* it will enrich genomic information since only a couple of transcriptome analyses have been reported.
• These data will be useful for studying genes differentially expressed in *Pistacia* species and useful for plant taxonomists to classify *Pistacia* species more accurately.
• These data can be used to identify compounds with pharmaceutical properties and to identify genes associated with synthetic pathways of the compounds of interest.
• Also, these datasets can be used to understand whether differentially expressed genes are related to distinctive therapeutic effects of each *Pistacia* species and related genera.

## 2 Background

*Pistacia chinensis* and *weinmannifolia* have long been used as important medicinal plants and are considered the best potential resources for developing new natural products for pharmaceutical purposes. However, genomic and genetic information are very limited for *P. chinensis* and not yet available for *P. weinmannifolia*. RNA-seq analysis was performed to provide genomic information of the two *Pistacia* species and also to characterize and compare differences in important genes, metabolic pathways, and networks associated with the production of secondary metabolites between the two species.

## 3 Data Description

**Fig. 1:**
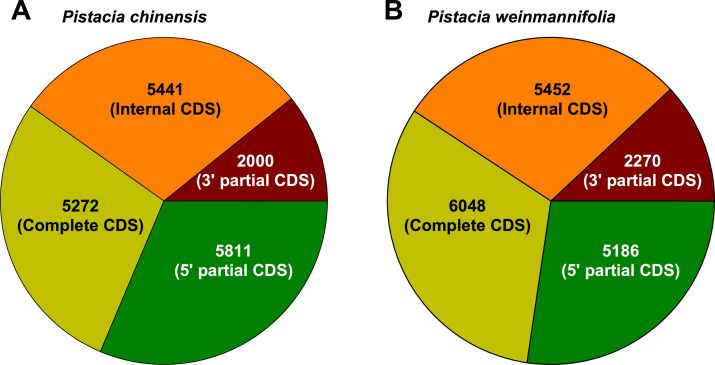
Characteristics of coding region of the contigs from transcriptome data of **A**) *Pistacia chinensis* and **B**) *Pistacia weinmannifolia*. Contigs designated to “complete” are potentially full length transcripts, whereas contigs with the “5′ partial”, “3′ partial”, and “Internal” are missing start codon, stop codon, or both in open reading frame.

**Fig. 2:**
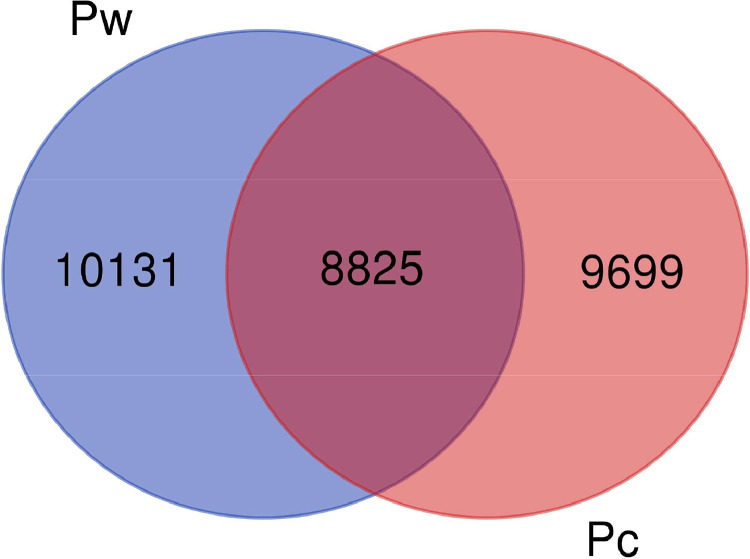
Comparison of contigs between *P. chinensis* (Pc) *and P. weinmannifolia* (Pw). Among all contigs, 8825 contigs were shown being likely homologous in two *Pistacia* transcriptome datasets.

The RNA sequencing data presented in this article are obtained from leaf samples of two different *Pistacia* species, *P. chinensis* and *P. weinmannifolia*. The raw RNA-seq data were deposited at NCBI Sequence Read Archive (SRA) database under the accession SRR10136265 for *P. chinensis* and SRR10136264 for *P. weinmannifolia*. This Transcriptome Shotgun Assembly (TSA) projects have been deposited at DDBJ/EMBL/GenBank under the accession GKQH00000000 for *P. chinensis* and GKQG00000000 for *P. weinmannifolia*. The TSA version described in this paper is the first version, GKQH01000000 and GKQG01000000. The raw and assembled RNA sequencing data are summarized in Table 1. The assembled contigs were annotated by running local BLAST against the NCBI non-redundant (NR) protein database. The assembled contigs showed that many of them contain only partial coding regions as shown in Fig. 1. Comparison of two transcriptomes showed that at least 8825 contigs are highly similar in these two transcriptomes (Fig. 2).

**Table 1:** Summary of raw and assembled sequence data.

Description of sequence data	Species	
	*Pistacia chinensis*	*Pistacia weinmannifolia*
Total number of raw reads	10,621,059	10,388,991
Total length of raw reads (bp)	2676,506,868	2618,025,732
Total number of filtered reads	9625,676	9161,308
GC content of raw reads (%)	40.7	41.3
Total length of filtered reads (bp)	2358,007,393	2230,762,660
Percentage of filtered quality reads	88 %	85 %
Number of assembled contigs	18,524	18,956
Number of annotated contigs	17,814	18,296
Total length of assembled contigs (bp)	16,174,683	17,080,830
Average length (bp)	873	901
Length of largest contig (bp)	9942	9783
N50 (bp)	1104	1137
GC content (%)	43.4	43.2

## 4 Experimental Design, Materials and Methods

### 4.1 Sample collection

Fresh leaves were collected from fully-grown *P. chinensis* and *P. weinmannifolia* in June 2015 at Yunnan, China. Leaf samples were submerged into liquid nitrogen, transferred into RNAlater solution for long-term transportation (Ambion Ins, USA), and then stored in −20 °C freezer until used for RNA extraction.

### 4.2 Transcriptome sequencing

Leaf samples removed from RNAlater solution were ground with a pestle and mortar in liquid nitrogen to extract total RNAs using TRIzol reagent (Thermo Fisher Scientific, Korea). Total RNAs were checked for quantity and purity using an RNA Pico Chip on the Agilent 2100 Bioanalyzer (Agilent Technologies, USA). A 10 μg of the total RNA was used for mRNA isolation using oligo-dT beads, the isolated mRNA was randomly sheared, and the adaptor was added by ligating to 3′ A overhang of the sheared mRNA for cDNA synthesis. The mRNA isolation and cDNA library construction was conducted by following the procedure of the SureSelect strand-specific RNA reagent kit (Agilent, USA). An equal quantity of mRNA from three different leaf samples was pooled and used for cDNA library construction. Quality check of the cDNA library was examined by Agilent DNA 1000 chip (Agilent Technologies, USA), and the Illumina Hiseq 2500 (Illumina, USA) was used for sequencing the cDNA library.

### 4.3 De novo assembly and annotation

The raw reads were trimmed and filtered to remove adaptor sequences, empty reads, and low quality reads with ≤20 of a phred quality score and ≤50 bp in length using NGS tool kits and Trimmomatic tool [2]. The filtered reads were assembled using three different assemblers, CLC Genomics Workbench (ver. 3.7.1), Trinity (release 20110519), and Velvet-Oases (ver. 1.1.04-ver. 0.1.21). A default k-mer value (25-mer) was used for the assembly with the CLC Genomics Workbench. For the assembly by Trinity and Velvet-Oases, different k-mer values (25–33 for Trinity; 21–79 for Velvet-Oases) were applied to obtain the best results. All contigs from each assembler were merged separately for further processing. As Oases does not cluster assembled contigs, CD-HIT-EST was used to cluster the contigs with an identity of more than 90 % and coverage of 100 % [3]. All data sets from each assembler were finally combined into a single dataset by collapsing identical contigs into a single contig using CD-HIT-EST with the same criteria described above. Contigs longer than 300 bp were annotated by running local BLAST with a cutoff E-value of 10^−6^ against the NCBI non-redundant (NR) protein database (http://www.ncbi.nlm.nih.gov).

## Limitations

None.

## Ethics Statement

The current work does not involve human subjects, animal experiments, or any data collected from social media platforms.

## CRediT authorship contribution statement

**Azeez Bimpe Suliyat:** Data curation, Writing – original draft. **Dewi Komang Anggita:** Data curation. **Hee Soo Yang:** Data curation. **Sang-Woo Lee:** Data curation, Resources. **Wan Yi Li:** Resources. **Sang-Ho Choi:** Data curation, Conceptualization. **Ki-Young Choi:** Writing – review & editing. **Jong-Kuk Na:** Conceptualization, Supervision.

## Data Availability

Transcriptome sequecing of two Pistacia species (Original data) (NCBI Bioproject)
